# Clinical effects of multi-strain probiotics in extremely preterm infants: a multicenter pre-post implementation study in the Netherlands

**DOI:** 10.1007/s00431-026-07560-8

**Published:** 2026-10-31

**Authors:** A. J. van Wesemael, R. R. de Kroon, L. M. H. E. van Eijk, N. M. Frerichs, M. J. Vermeulen, C. H. P. van den Akker, M. M. A. Raets, C. V. Hulzebos, W. P. de Boode, A. H. van Kaam, T. G. J. de Meij, H. J. Niemarkt

**Affiliations:** 1 Department of Pediatric Gastroenterology, Emma Children’s Hospital, Amsterdam UMC, Amsterdam, Netherlands; 2 Amsterdam Reproduction and Development Research Institute, Amsterdam UMC, Amsterdam, Netherlands; 3 Amsterdam Gastroenterology Endocrinology & Metabolism Research Institute, Amsterdam UMC, Amsterdam, Netherlands; 4 Department of Pediatrics, Catharina Hospital, Eindhoven, Netherlands; 5 Department of Neonatal and Pediatric Intensive Care, Division of Neonatology, Sophia Children’s Hospital, Erasmus MC, Rotterdam, Netherlands; 6 Department of Pediatrics - Neonatology, Emma Children’s Hospital, Amsterdam UMC, University of Amsterdam, Amsterdam, Netherlands; 7 Department of Neonatology, MosaKids Children’s Hospital, Maastricht UMC, Maastricht, Netherlands; 8 Department of Neonatology, Beatrix Children’s Hospital, UMC Groningen, Groningen, Netherlands; 9 Department of Neonatology, Amalia Children’s Hospital, Radboud UMC, Nijmegen, Netherlands; 10 Department of Neonatology, Maxima Medical Centre, Veldhoven, Netherlands; 11 Department of Electrical Engineering, Technical University Eindhoven, Eindhoven, Netherlands

**Keywords:** Microbiome, Premature infant, Necrotizing enterocolitis, Late-onset sepsis, Postnatal growth, Bifidobacteria

## Abstract

Probiotics may reduce the risk of necrotizing enterocolitis (NEC), late-onset sepsis (LOS), and mortality in preterm infants. However, their efficacy in extremely preterm infants (gestational age < 28 weeks) is still regularly questioned due to limited data and safety concerns. In Dutch Neonatal Intensive Care Units (NICUs), a three-strain probiotic product (ProPrems®; *Bifidobacterium infantis* Bb-02, *Bifidobacterium lactis* BB-12, and S*treptococcus thermophilus* TH-4) was implemented as standard of care from 2021 onwards. This study evaluates the clinical effects of these probiotics in extremely preterm infants. Pre- and postimplementation cohorts of extremely preterm infants from 6 Dutch NICUs (2018–2023) were compared. Clinical data from the first 29 days of life were collected. Incidence of NEC (modified Bell’s stage ≥ IIA), LOS, and mortality were compared with logistic regression; growth velocity with linear regression; and time to full enteral feeds (tFEF) with survival analyses. In total, 481 extremely preterm infants (212 pre-, 269 post-implementation) were included. After implementation, the adjusted odds ratio was 0.49 (0.25–0.99; *p* = 0.049) for NEC; 0.87 (0.59–1.28; *p* = 0.49) for LOS; and 0.67 (0.35–1.27; *p* = 0.22) for all-cause mortality. Furthermore, tFEF decreased by 2 days (*p* < 0.001) and probiotic use was associated with an increase in growth velocity of 1.8 g/kg/day (*p* < 0.001). One infant (0.4%) developed probiotic-associated sepsis. *Conclusion*: This multicenter pre-postimplementation study showed that multi-strain probiotic administration was associated with lower NEC incidence, shorter tFEF, and greater growth velocity in extremely preterm infants during the first four weeks of life. Probiotic-associated sepsis was rare. These results support continued use of probiotics in this vulnerable population.

**Table:** 

**What is Known:** *• Certain probiotic strains effectively reduce incidence of necrotizing enterocolitis (NEC) in preterm infants, but the efficacy and safety for extremely preterm infants (GA < 28 weeks) remain under debate.*	
**What is New:** *• This study shows that a multi-strain probiotic supplement was associated with lower NEC incidence, shorter time to full enteral feeds, and greater early postnatal growth in a Dutch multicenter cohort of extremely preterm infants.* *• The incidence of probiotic-associated sepsis in extremely preterm infants was very low in our cohort with just 1 case (0.4%), consistent with the rare occurrence reported previously.*

**Supplementary Information:**

The online version contains supplementary material available at https://doi.org/10.1007/s00431-026-07560-8.

## Introduction

The administration of enteral probiotics in preterm infants with a gestational age (GA) < 32 weeks has increasingly been implemented as standard of care in neonatal intensive care units (NICUs) worldwide [1]. Numerous studies have shown that supplementation of certain probiotic strains in this vulnerable population is associated with a reduced incidence of necrotizing enterocolitis (NEC) and may also contribute to lower incidences of late-onset sepsis (LOS) and all-cause mortality [2, 3]. Additionally, probiotics have been linked to improved feeding tolerance and postnatal growth and reduced duration of parenteral nutrition dependency [4, 5]. In light of these benefits, and following international recommendations [6], the use of a three-strain probiotic supplement containing *Bifidobacterium animalis* subsp. *lactis* BB-12*®*, *Bifidobacterium longum* subsp. *infantis* Bb-02, and *Streptococcus thermophilus* TH-4*®* (ProPrems*®*, Neobiomics, Sweden) was recommended as routine care in the Netherlands in 2021 and was implemented across the Dutch NICUs at various timepoints over the past years.

The preterm gut differs significantly from that of term infants, with increased dysmotility, impaired mucosal integrity, higher intestinal permeability, and an altered microbiota composition [7]. Compared to term infants, the preterm microbiota shows reduced levels of beneficial bacteria such as Bifidobacterium and increased colonization by potentially pathogenic genera like *Enterococcus* and *Klebsiella* [8]. Probiotics are thought to benefit preterm infants primarily by modulating the gut microbiota and by postbiotic effects which enhance gut maturation, immune development, and gut barrier function through multiple pathways [9].

Although certain probiotic formulations have been shown to improve important clinical outcomes in very preterm infants, uncertainty remains regarding their efficacy and safety in extremely preterm infants (GA < 28 weeks), despite this group being at the highest risk for NEC, LOS, and feeding difficulties [10, 11]. This debate stems primarily from the underrepresentation of extremely preterm infants in most clinical trials, resulting in studies that lack sufficient statistical power to detect meaningful effects in this sub-population [2, 3]. In addition, their immature intestinal barrier and immune system may increase susceptibility to rare adverse events such as probiotic-associated sepsis [12, 13]. These safety concerns, reinforced by a recent U.S. Food and Drug Administration warning, have contributed to changes in clinical practice and renewed debate regarding routine probiotic supplementation [14–17].

Moreover, existing studies vary considerably with respect to probiotic strains, dosing regimens, treatment duration, and study populations, limiting the generalizability of their findings [3]. While randomized controlled trials establish efficacy under controlled conditions, real-world implementation studies are essential to evaluate effectiveness in conditions that more closely resemble everyday neonatal care. However, such studies remain scarce and have generally been limited to single centers, mixed gestational-age populations, and probiotic formulations that differ from those currently recommended in international guidelines [18].

The nationwide implementation of ProPrems® across Dutch NICUs provided a unique opportunity to generate real-world evidence on the effectiveness of routine probiotic supplementation while focusing specifically on extremely preterm infants. Therefore, this multicenter retrospective pre–post implementation study evaluated the association between routine multi-strain probiotic supplementation and clinically relevant outcomes, including NEC, LOS, all-cause mortality, enteral feeding advancement, and postnatal growth during the first month of life.

## Methods

### Study design and participants

This cohort study on the efficacy of probiotic supplementation was embedded in an ongoing multicenter observational cohort study in 8 Dutch NICUs, aimed at identifying fecal biomarkers for NEC and LOS in extremely preterm infants in the first 29 days of life. It was initiated in 2014 and approved by each of the local medical ethical review boards (protocol 2013.386, amendment A2016.363).

In the current embedded cohort study, all live-born infants with a GA > 24 weeks and < 28 weeks without major gastrointestinal congenital anomalies were eligible for inclusion if they were born during the pre- and postimplementation cohorts with equal study period duration, so that the length of inclusion differed for each center. Two out of the 8 centers from the overarching fecal biomarker study did not recruit during both the pre- and postimplementation periods and were therefore excluded from this study. Two other centers (3 and 4) temporarily dropped out of the study for a period of time, due to the COVID-19 pandemic, resulting in non-continuous pre/post-periods. Included infants who died or were transferred to a non-participating NICU within the first 72 h of life were excluded (*n* = 1). Table 1 shows an overview of study participants and inclusion periods per center. It must be noted that infants from Center 1 were also included in a previously published study on the association between probiotic implementation and NEC in infants born < 30 weeks gestation or with birth weight < 1000 g [19]; they represent a subgroup born < 28 weeks’ gestation within that cohort. These infants were retained because the present study addresses a different research question, using a multicenter design across six NICUs and additional outcomes including time to full enteral feeding and postnatal growth.

**Table 1:** Overview of study participants and inclusion periods per center

NICU	Pre-implementation epoch	Infants (pre)	Implementation start	Post-implementation epoch	Infants (post)	Dropout period
1	Aug 2018–Feb 2021	77	Mar 2021	Mar 2021–Sep 2023	120	-
2	Jun 2020–Feb 2021	14	Mar 2021	Mar 2021–Nov 2021	25	-
3	Mar 2018–Aug 2019	69	Nov 2021	Jul 2022–Dec 2023	53	Sep 2019–Jun 2022
4	May 2019–May 2020	12	Jan 2022	Oct 2022–Oct 2023	26	Jun 2020–Sep 2022
5	May 2021–Jan 2022	20	Feb 2022	Feb 2022–Oct 2022 (24)	24	-
6	Jul 2022–Feb 2023	20	Mar 2023	Mar 2023–Oct 2023	21	-
Total		212			269	

### Probiotic administration and enteral feeding practices

Infants received the three-strain probiotic mixture (ProPrems*®*, *Neobiomics*, *Sweden*) according to nationwide recommendation guidelines. The three strains were *Bifidobacterium animalis* subsp. *lactis* BB-12*®* (1.8 * 10^9^ CFU), *Bifidobacterium longum* subsp. *infantis* Bb-02 (DSM33361; 1.8 * 10^9^ CFU), and *Streptococcus thermophilus* TH-4*®* (1.5 * 10^9^ CFU). The probiotics were administered once daily in all infants with a GA < 30 weeks and/or a birth weight < 1000 g admitted to the NICU, with a minimal postnatal age of 48 h and a minimum requirement of tolerating 2 mL per feeding. Contraindications were minimal enteral feeding, nil per os, or severe gastrointestinal disease where the gut integrity was considered compromised, such as NEC or congenital gastrointestinal anomalies. Probiotics were administered until discharge from the NICU or a postmenstrual age of 35 weeks.

Infant’s nutrition was based on local protocols, which varied among the NICUs. All centers prioritized fresh mother’s own milk (MOM) and all but one center (center 5) had access to pasteurized donor human milk (DHM) throughout the study period. Center 5 had DHM available 3 months after the start of the pre-implementation cohort. All centers applied fortification of human milk with breast milk fortifier and modular protein or lipids.

### Data collection

Clinical data were collected throughout the first 29 days of life. Clinical data collected included demographic and perinatal characteristics; medication use (including antibiotics, probiotics, inotropes, corticosteroids, non-steroidal anti-inflammatory drugs (NSAIDs); feeding characteristics (type and volume); respiratory support (type and duration); clinical outcomes (NEC, focal intestinal perforation, LOS, mortality); time to discharge from NICU; and growth measured at postnatal days 7, 14, 21, or 28 (± 3 days).

### Outcome variables and definitions

The outcomes of this study were the incidence and severity of NEC, LOS, all-cause mortality, time to full enteral feeding (tFEF), and postnatal growth, all assessed in the first 29 days of life. NEC was defined as a modified Bell’s stage IIA or higher [20], where the staging was assigned after thorough chart- and radiology review by two clinical experts (HN and TdM). Surgical NEC included NEC cases that were surgically managed (through surgery or peritoneal drain placement) and infants who died before surgical intervention could take place. NEC-related mortality was defined as mortality throughout the time period the infant was treated for the NEC episode.

Culture-proven LOS was defined as a bacterium or fungus isolated from blood and/or cerebrospinal fluid, obtained > 3 days after birth [21]. An exception was made for LOS caused by coagulase-negative *Staphylococci* (CoNS), where the used definition was derived from the national recommendation, stating (1) clinical signs of infection > 72 h after birth and (2) blood culture solely positive for CoNS and (3) C-reactive protein (CRP) > 10 mg/L within 24–36 h after the initial sepsis evaluation [22]. Clinical LOS was defined as (1) clinical signs of infection > 72 h after birth and (2) negative blood and CSF culture and (3) CRP > 10 mg/L within 24–36 h after the initial sepsis evaluation and (4) the intention to treat for ≥ 5 consecutive days with antibiotic therapy. tFEF was defined as the day of life when parenteral nutrition, including parenteral glucose, was stopped, where the day of birth is counted as day 0. Type of feeding was assessed at tFEF, or—if tFEF never occurred—at the last known day that enteral feeds were consumed. Growth velocity was calculated using the Patel exponential model between birth weight and last-known weight within the first 29 days of life [23]. Growth Z-scores and percentiles were calculated using the Fenton growth charts for preterm infants [24].

### Safety

Safety of the implementation of probiotics was assessed by the incidence of probiotic-associated sepsis, defined as an administered probiotic strain cultured from blood or CSF.

### Statistical analysis

Demographics of the included population were analyzed using descriptive statistics and frequencies in IBM SPSS statistics 28. The pre- and postimplementation cohorts were compared with a *t*-test or Mann–Whitney *U* for continuous outcomes, depending on normality of the data, and the chi-square test or Fisher’s exact test for categorical outcomes. A logistic regression model was used to calculate (adjusted) odds ratios (ORs) for the main disease outcomes. For NEC, LOS, and mortality, the logistic regression model was adjusted for birth weight, given its known association to the outcomes of interest. No further variables were included due to the limited number of cases and associated risk of overfitting. To assess tFEF, Kaplan–Meier survival analysis was performed, censoring infants who never reached tFEF throughout the study period. A time-dependent Cox proportional hazards model was used to adjust for birth weight; type of feeding (MOM, DHM, or formula); and a time-dependent composite variable for NEC, culture-proven LOS, and/or mortality. A multivariate linear model was employed to investigate the effect of probiotics on growth velocity, adjusting for type of feeding at full enteral feeding, being born small-, appropriate-, or large for gestational age (SGA, AGA, or LGA); biological sex; and a composite variable including NEC, culture-proven LOS, or mortality. The assumptions of linear regression were assessed using residual diagnostics. *p*-values < 0.05 were considered statistically significant. The survival analyses and multivariate linear model were performed using the “lme4,” “stats,” “survival,” and “survminer” packages from R version 4.5.1.

## Results

### Baseline and clinical characteristics

In total, 481 infants were included, of whom 269 during the post-probiotics epoch. The mean gestational age was 26 3/7 weeks. All infants in the post-implementation epoch received probiotics during the first 29 days of life. The median postnatal day of initiation of probiotics supplementation was 3 (interquartile range (IQR) 1; minimum 0; maximum 24). The median duration of probiotic supplementation was 25 days (IQR 7; minimum 1; maximum 28). Between the two groups, there were significant differences in the type of feeding, NSAID use for patent ductus arteriosus, and infants who received a red blood cell transfusion during the first 29 days of life (Table 2).

**Table 2:** Baseline and clinical characteristics of included infants

	Pre-implementation epoch (*n* = 212)	Post-implementation epoch (*n* = 269)	*p*-value
Gestational age [wks + d; mean (SD)]	26 + 2 (8)	26 + 4 (7)	0.096
Birth weight [g; mean (SD)]	868 (204)	893 (194)	0.180
SGA [*n* (%)]	23 (11)	25 (9)	0.707
Biological sex, female [*n* (%)]	102 (48)	111 (41)	0.525
Singleton [*n* (%)]	151 (71)	182 (67)	0.400
Vaginal delivery [*n* (%)]	119 (56)	131 (49)	0.094
Infectious pregnancy complication [*n* (%)]	98 (46)	113 (42)	0.355
Hypertensive pregnancy complication [*n* (%)]	50 (24)	58 (22)	0.597
Apgar score < 7 at 5 min [*n* (%)]	66 (31)	67 (25)	0.132
Type of feeding^†^ [*n* (%)]			< 0.001
- Predominant MOM - Predominant DHM - Predominant formula	180 (85) 15 (7) 15 (7)	223 (83) 43 (16) 0 (0)	
Days of antibiotic treatment in first 29 days of life [median (IQR)]	7 (10)	6 (8)	0.093
Empiric antibiotic treatment for suspected EOS [*n* (%)]	176 (83)	224 (83)	0.941
Inotropes [*n* (%)]	67 (32)	72 (27)	0.252
Corticosteroid use for BPD prevention [*n* (%)]	36 (17)	61 (23)	0.122
NSAID use for PDA [*n* (%)]	77 (36)	37 (14)	< 0.001
Surfactant [*n* (%)]	155 (73)	195 (72)	0.852
RBC transfusion [*n* (%)]	149 (70)	137 (51)	< 0.001
Duration of NICU admittance [median (IQR)]	37 (32)	38 (25)	0.933

Infectious pregnancy complications include prolonged rupture of the membranes, maternal sepsis or infection, maternal fever during labor, chorioamnionitis, and/or group B Streptococcus carriage. Hypertensive pregnancy complications include pre-eclampsia, hypertension, HELLP syndrome, and/or intrauterine growth restriction

*SGA*, small for gestational age, birthweight < 10th percentile as assessed with the Fenton Prematurity Growth Chart 2013; *EOS*, early onset sepsis; *BPD*, bronchopulmonary dysplasia; *NSAID*, nonsteroidal anti-inflammatory drugs; *PDA*, patent ductus arteriosus; *RBC*, red blood cell

^†^Defined as > 50% of enteral volume at reaching FEF or last day of admittance when sooner

^*^Defined as the cumulative days of iv antibiotic treatment divided by the total admission days in the first 29 days of life

### Implementation of probiotics is associated with a reduction in NEC

**Fig. 1:**
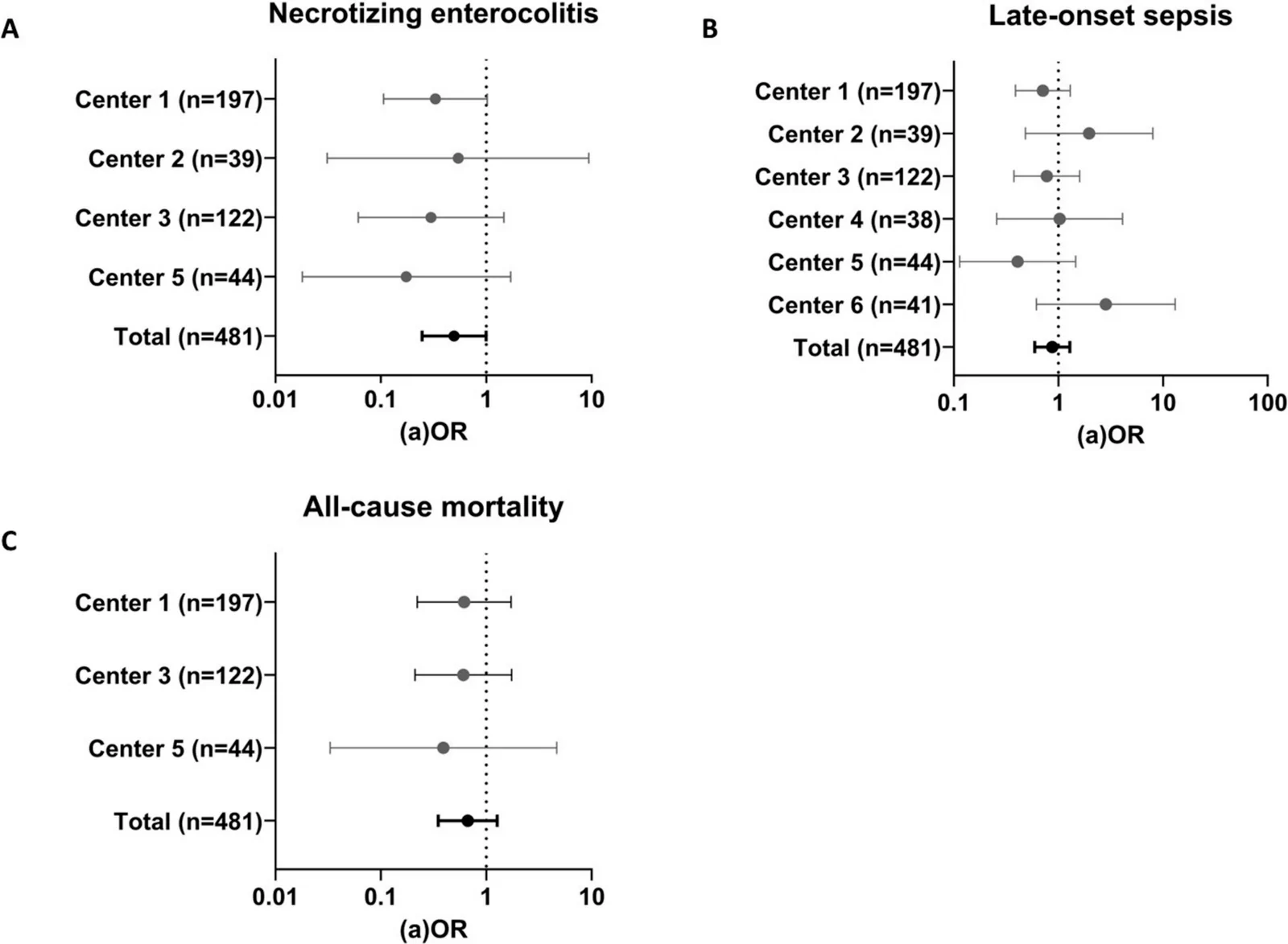
Forest plot of odds ratios per center of birth for NEC (**A**), LOS (**B**), and mortality (**C**). Per center, the crude OR is shown, for all centers combined (total), the aOR is shown, adjusted for birth weight. Centers 4 and 6 are missing from panel (**A**), due to all NEC cases in those centers being in the post-implementation cohort. The data from these centers is included in the Total aOR in the figure. Centers 2, 4, and 6 are missing from panel (**C**), due to all mortality cases being in either the pre- or post-implementation cohort. The data from these centers is included in the Total aOR in the figure. This figure was created using GraphPad Prism 10

The incidence of NEC (Bell’s stage ≥ IIA) was lower in the post-implementation epoch compared to the pre-implementation epoch with an aOR of 0.49 (95% CI: 0.25–0.99, Table 3, and Fig. 1A). The absolute risk reduction was 5%, resulting in a number needed to treat of 20 (95% CI 10–323) to prevent one additional case of NEC. This difference in incidence is largely explained by the difference in medical NEC. Figure 1a depicts the (a)OR for NEC per NICU, showing a consistent trend in all participating centers which encountered sufficient NEC cases to calculate ORs. In a sensitivity analysis additionally adjusting for formula feeding, the association between probiotic supplementation and reduced NEC incidence remained statistically significant (aOR 0.46, *p* = 0.042).

**Table 3:** Incidence and adjusted odds ratios of NEC, LOS, FIP, and mortality stratified by probiotic exposure

	Pre-implementation epoch (*n* = 212)	Post-implementation epoch (*n* = 269)	*p*-value	aOR (95% CI)	*p*-value
NEC [*n* (%)] ⚬ Medical ⚬ Surgical ⚬ 2A/B ⚬ 3A/B NEC onset DoL, median (IQR) NEC-related mortality, *n* (%) Concurrent LOS, *n* (%)	22 (10) 9 (4) 13 (6) 7 (3) 15 (7) 12 (11) 9 (41) 14 (64)	14 (5) 4 (1) 10 (4) 3 (1) 11 (4) 9 (9) 8 (57) 10 (71)	0.032 0.317 0.342 0.629	0.49 (0.25–0.99)	0.049
LOS [*n* (%)] Culture proven - CoNS * - Staphylococcus aureus* - Non-staphylococcal pathogens^#^ Clinical sepsis	83 (39) 69 (33) 35 (17) 12 (6) 23 (11) 16 (8)	93 (35) 78 (29) 40 (15) 15 (6) 25 (9) 19 (7)	0.301 0.401 0.623 0.968 0.572 0.975	0.87 (0.59–1.28) 0.89 (0.60–1.33)	0.488 0.572
FIP [*n* (%)]	8 (4)	3 (1)	0.053	n.a	n.a
All-cause mortality [*n* (%)]	23 (11)	19 (7)	0.144	0.67 (0.35–1.27)	0.218
Composite NEC, culture-proven LOS and mortality	82 (39)	85 (32)	0.105	0.77 (0.52–1.13)	0.176

All reported incidences are within the first 29 days of life

*NEC *necrotizing enterocolitis, *Surgical NEC *surgically treated or NEC-related death, *DoL* day of life, *LOS *late-onset sepsis, *FIP *focal intestinal perforation, *aOR* adjusted odds ratio. Odds ratios were adjusted for birth weight

^#^The “non-staphylococcal pathogens” subgroup includes *E. coli*, *Candida* spp., *Enterobacter* spp., *Enterococcus* spp., group-B streptococcal species, *Pseudomonas* spp., *Klebsiella* spp., *Serratia* spp. and *Citrobacter* spp.

No statistically significant differences in the incidence of LOS, NEC-related mortality or all-cause mortality were observed in the first 29 days of life. When stratifying LOS cases based on the causative pathogen, no significant differences were seen. Figure 1B–C shows the (a)OR for LOS and mortality per NICU.

One infant presented with probiotic-associated LOS with *B. infantis* (confirmed after whole genome sequencing of the cultured organism) during a concurrent fulminant NEC episode after which the infant died. This resulted in an incidence of 0.4% for probiotic-associated sepsis in our cohort.

### Implementation of probiotics is associated with a reduction of tFEF and increased early postnatal growth

**Fig. 2:**
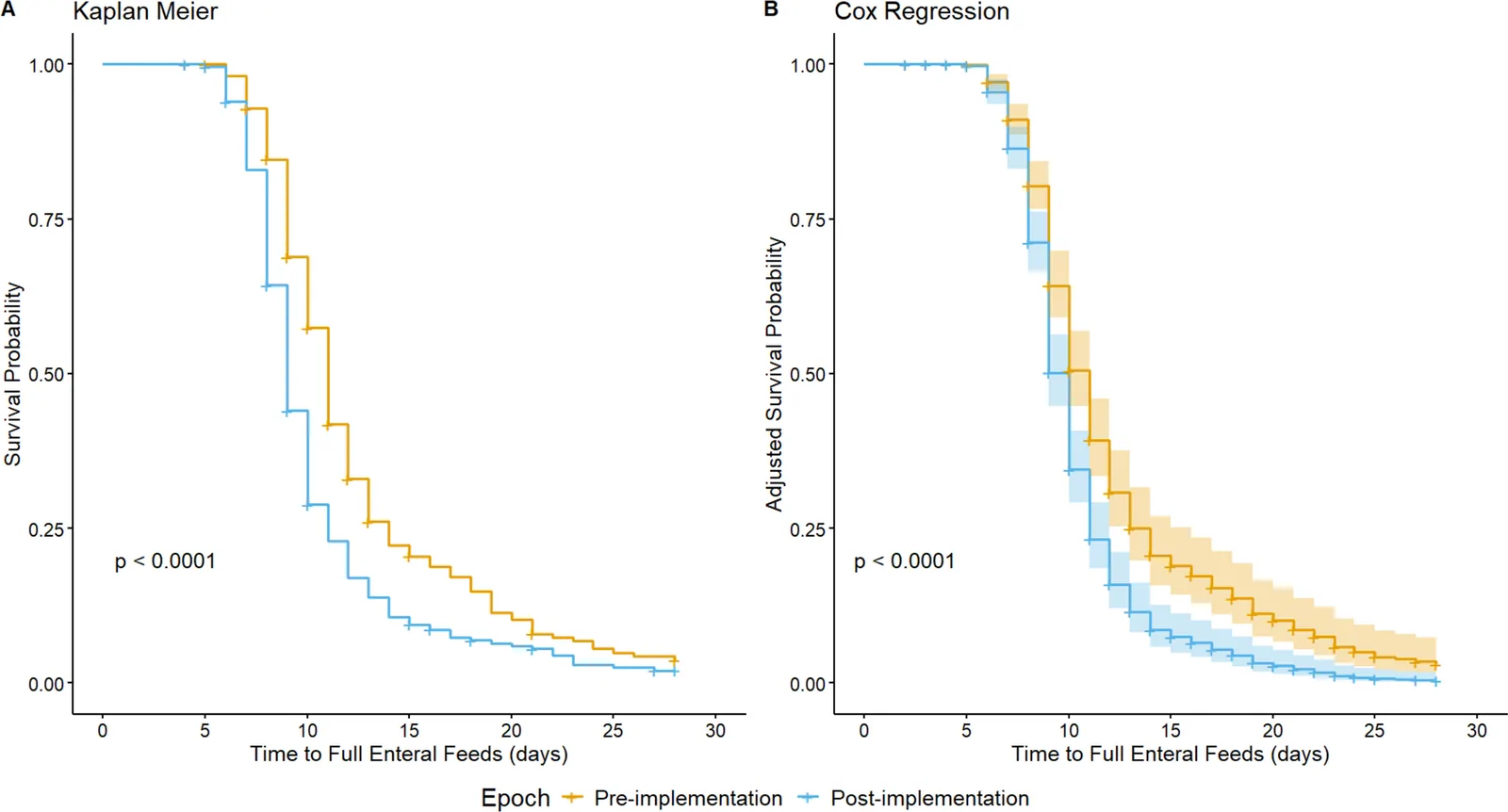
**A** The Kaplan–Meier curve for time from birth to full enteral feeds, stratified by epoch. The *p*-value represents the log-rank test result. **B** The adjusted survival curve for time from birth to full enteral feeds, stratified by epoch, from a time-dependent multivariate Cox proportional hazards model, holding covariates at sample means or modal values. The model is adjusted for birth weight, a composite outcome of NEC, culture-proven LOS and/or mortality (time-dependent), and type of feeding. The *p*-value reflects the adjusted association between epoch and time to full enteral feeds, based on the Wald test. This figure was created using R 4.3.2

Table 4 presents feeding practices, feeding outcomes, and growth parameters not previously reported in Table 2. Kaplan–Meier survival analysis, where infants who never received FEF due to mortality or discharge were censored, confirmed 2 days earlier FEF after routine implementation of probiotics (median tFEF of *11 days; 95% CI 10.6*–*11.4* for the pre-implementation epoch; median tFEF of *9 days; 95% CI 8.7*–*9.3* for the post-implementation epoch; *p* < *0.0001;* Fig. 2A). In a multivariate time-dependent Cox proportional hazards model, adjusted for birth weight, a composite outcome of NEC, culture-proven LOS and/or mortality (time-dependent), and type of feeding, the use of probiotics was associated with a significantly earlier tFEF (aHR: *1.5; 95% CI 1.2*–*1.8; p* < *0.0001*; Supplemental table S1 and Fig. 2B).

**Table 4:** Feeding and growth characteristics and outcomes

	Pre-implementation epoch (*n* = 212)	Post-implementation epoch (*n* = 269)	*p*-value
Feeding			
Reached FEF within first 29 days* [*n* (%)]	188 (88)	251 (95)	0.074
Enteral volume at FEF, mL/kg/day [mean (SD)]	155 (14)	156 (16)	0.627
tFEF, days [median (IQR)]	11 (4)	9 (3)	< 0.001
Ever received DHM [*n* (%)]	100 (47)	216 (80)	< 0.001
Ever received formula [*n* (%)]	89 (42)	40 (15)	< 0.001
BMF addition [*n* (%)]	199 (96)	255 (95)	0.435
Modular protein addition [*n* (%)]	88 (42)	86 (32)	0.017
Modular lipid addition [*n* (%)]	33 (16)	59 (22)	0.102
Growth			
Weight Z-score DoL 28 [mean (SD)]	− 0.8 (0.6)	− 0.7 (0.6)	0.032
Weight Z-score last known weight [mean (SD)]	− 0.7 (0.6)	− 0.6 (0.6)	0.112
ΔZ-score between birth weight and weight DoL 28 [mean (SD)]	− 0.7 (0.5)	− 0.7 (0.5)	0.847
ΔZ-score between birth weight and weight D28 or discharge or death when sooner [mean (SD)]	− 0.8 (0.5)	− 0.7 (0.6)	0.375
Growth velocity** [mean (SD)]	9.6 (5.7)	11.4 (4.3)	< 0.001

*tFEF* time to full enteral feeding, *MOM* mother’s own milk, *DHM* donor human milk, *BMF* breast milk fortifier

*Or up until discharge or death when sooner

**According to the Patel exponential model, from birth to DOL28

In a multivariate linear regression model, probiotic use was associated with significantly greater average growth velocity from birth to DOL 28 (adjusted mean difference: 1.8 g/kg/day, 95% CI 0.9 to 2.7, *p* < 0.0001*)* (Supplemental tableS2). Infants who encountered any of the composite adverse outcomes (NEC, LOS, or mortality), who received predominantly DHM, or who were not SGA had significantly lower growth velocities.

## Discussion

In this multicenter retrospective study, we observed that administration of a multi-strain probiotic product (ProPrems*®*) to extremely preterm infants was associated with a significant reduction in the incidence of NEC, shorter tFEF, and improved early postnatal growth in the first month of life. Importantly, the observed reduction in NEC was consistent across participating centers, and the beneficial effects on feeding and growth were supported by both unadjusted and multivariate analyses. These findings suggest that probiotic supplementation provides meaningful clinical benefits in this highest-risk subgroup of extremely preterm infants.

The observed halving of NEC incidence in extremely preterm infants is in line with the current literature on its efficacy in very preterm infants born between 28 and 32 weeks of gestation [2, 25]. Noteworthy, several studies did not find statistically significant differences in NEC incidence in a subgroup of extremely preterm infants, including the original ProPrems Trial (2013), that investigated the same strains as studied here [26]. However, these studies often lacked sufficient statistical power to detect differences in this subgroup or were primarily designed and powered to assess LOS, instead of NEC. We found the largest NEC reduction in infants with the relatively milder forms of medically managed NEC, which may explain the absence of a decrease in NEC-related and all-cause mortality in the post-implementation epoch.

Our data show no differences in LOS incidence between pre- and post-implementation periods. To explore potential differences according to presumed etiology (gut versus skin or invasive devices), we categorized LOS cases by pathogen (CoNS, *S. aureus*, and non-staphylococcal) [27, 28]. In line with the ProPrems trial and the subgroup analyses on extremely preterm infants of a Cochrane review [2, 26], we found no reductions in pathogen-specific subgroups.

In addition to the aforementioned disease-outcomes, our results showed that infants receiving probiotics reach FEF 2 days sooner than infants who do not receive probiotics, which was statistically significant independent of birth weight, type of feeding and the occurrence of NEC, culture-proven LOS, and/or mortality. This is in line with previous data in preterm infants, showing a mean difference of − 1.5 days, and complements a recent systematic review, where a non-significant reduction of 2.4 days was observed in extremely preterm infants [29]. Mechanisms that may underlie the improved tFEF in probiotic-supplemented infants include enhancement of gastrointestinal motility through microbial metabolite signaling, such as increased gut-derived serotonin production [30, 31]. In addition, probiotics may reduce intestinal inflammation, modulate cytokines that influence gut neuromuscular function, and support mucosal barrier integrity, all potentially contributing to more rapid progression to FEF [31–33].

This faster advancement to FEF might contribute to our finding that growth velocity within the first month of life was significantly higher in infants who received probiotics. We consider the increase of 1.8 g/kg/day to be clinically relevant, as improvements in early weight gain have been associated with better neurodevelopmental outcomes [34]. However, this finding must be interpreted with caution, as other analyzed growth outcomes, such as the ΔZ-score between birth and last known weight within the first 29 days of life, showed a non-significant decrease. This may be attributed to increased use of donor milk or the more technical fact that the Patel exponential model accounts for both the infant’s size and the exact time between weights, without assuming linear growth between two time points, providing a more accurate daily growth rate [23, 35].

Rare cases of probiotic-associated sepsis in extremely preterm infants have been documented, inciting safety concerns of its use in this vulnerable population [12, 13, 16]. In our cohort with extremely preterm neonates, one infant developed a probiotic-associated sepsis during a concurrent NEC episode. While this case underscores that translocation of probiotic organisms can occur in the context in of severe intestinal mucosal injury, it also highlights the rarity of such events relative to the overall observed benefits. This is further underlined by a recent meta-analysis, stating that to prevent one case of probiotic-associated sepsis, an estimated 62 additional cases of NEC, 42 deaths, and 92 clinical sepsis events would occur amongst probiotic-unexposed preterm infants [12]. These findings support the continued use of effective probiotic strains in extremely preterm infants, provided that clinical protocols incorporate monitoring and robust safety measures and that the probiotic species are sensitive to the empirical antibiotic treatment of choice [6].

Strengths of this study include the multi-center design, the relatively large sample size of exclusively extremely preterm infants, and the consistent use of the one probiotic product and clinical protocol across all participating NICUs, allowing to perform pooled analysis of the outcomes. Furthermore, it adds to the available evidence on the efficacy of the multistrain probiotic supplement used in the Netherlands, with this study being the second to describe statistically significant beneficial effects in the extremely preterm population [19]. However, several limitations should be acknowledged. First, the observational design and pre-post comparison may have introduced bias, making it difficult to isolate the effects of probiotics from other temporal factors or secular trends. Additionally, data collection was limited to the first 29 days of life, which may have resulted in underreporting of NEC, LOS, or mortality occurring beyond this period. Non-adjacent study periods in two centers, partly due to the COVID-19 pandemic, raise concerns about time-related changes in clinical practice that could have influenced the outcomes of interest. For example, red blood cell transfusions and NSAID use were less common in the post-implementation cohort. While these factors have been suggested to influence NEC risk, existing evidence remains inconclusive, with two recent RCTs on PDA treatment and one on red blood cell transfusions showing no clear effect on NEC incidence [36–39]. Additionally, a subset of infants included in this study (center 1) has also been described previously as part of another cohort [19]. The present analysis, however, focuses exclusively on extremely preterm infants and incorporates additional outcomes (growth and feeding), multivariable analyses, and data from additional Dutch NICUs. Additionally, a post hoc sensitivity analysis excluding infants from center 1 showed a similar reduction on the primary outcome (NEC OR 0.60, 95% CI:0.25–1.46). Furthermore, the consistency of observed effect on NEC across the different Dutch NICUs (Fig. 1A), argues against findings being attributable to center-specific practices alone and supports the generalizability of results within the Dutch NICU setting.

Although the overall sample size was relatively large and pooling data from multiple centers was feasible due to the consistent probiotic formulation, stratified analyses per individual NICU were limited by small sample sizes in some units. The upcoming PEPS trial, a large (*n* = 1620) multicenter RCT assessing the same multistrain product in infants < 28 weeks gestation, is expected to overcome limitations inherent to observational before-and-after designs [40].

Beyond this, future research should extend follow-up durations to include longer-term outcomes and explore the underlying biological mechanisms through which probiotics exert their benefits. Understanding these mechanisms could help identify which infant subgroups benefit most, guide optimized timing, dosing and duration, and support the development of targeted or sterile formulations, such as postbiotics.

In conclusion, we demonstrated that in a real-world setting, a multi-strain probiotic supplement was associated with a reduction in NEC incidence, shorter tFEF, and increased postnatal growth velocity in infants born extremely preterm. Probiotic-associated sepsis was rare. This supports the use of probiotics in infants born < 28 weeks of gestation, extending evidence that has so far largely derived from more mature preterm populations.

## Supplementary Information

Below is the link to the electronic supplementary material.

ESM 1 (DOCX 24.4 KB)

## Data Availability

The datasets generated and analyzed during the current study are not publicly available due to ethical and privacy reasons, but are available from the corresponding author on reasonable request.

## Abbreviations

AGA: Appropriate for gestational age
aOR: Adjusted odds ratio
BMF: Breast milk fortifier
BPD: Bronchopulmonary dysplasia
CI: Confidence interval
CoNS: Coagulase-negative staphylococci
CRP: C-reactive protein
CSF: Cerebrospinal fluid
DHM: Donor human milk
DoL: Day of life
EOS: Early onset sepsis
FEF: Full enteral feeding
FIP: Focal intestinal perforation
GA: Gestational age
HELLP: Hemolysis, elevated liver enzymes, low platelets
IQR: Interquartile range
LGA: Large for gestational age
LOS: Late-onset sepsis
MOM: Mother's own milk
NEC: Necrotizing enterocolitis
NICU: Neonatal intensive care unit
NSAID: Non-steroidal anti-inflammatory drug
OR: Odds ratio
RBC: Red blood cell
SGA: Small for gestational age
tFEF: Time to full enteral feeding
