# Supplementary material for: Clinical effects of multi-strain probiotics in extremely preterm infants: a multicenter pre-post implementation study in the Netherlands
Source: Eur J Pediatr. 2026 Oct 31;185(11):913. doi: 10.1007/s00431-026-07560-8 (PMC13722641; doi:10.1007/s00431-026-07560-8)
Supplement: Supplementary file 1 [file 431_2026_7560_MOESM1_ESM.docx]

**Supplemental material**

Corresponding manuscript title: “Clinical effects of multi-strain probiotics in extremely preterm infants: a multicenter pre-post implementation study in the Netherlands”

**Supplemental table S1.** Time dependent Cox proportional hazards model on time to full enteral feeds.

| **Predictor** | **HR (exp(β))** | **95% CI (HR)** | **p-value** |
| --- | --- | --- | --- |
| **Probiotics (Yes vs No)** | 1.56 | [1.28, 1.90] | <0.001 |
| **Birthweight (per gram)** | 1.002 | [1.001, 1.002] | <0.001 |
| **Donor Human Milk (vs MOM)** | 1.07 | [0.80, 1.43] | 0.664 |
| **Formula (vs MOM)** | 0.83 | [0.46, 1.50] | 0.546 |
| **Time-dependent: NEC/LOS/mortality (yes vs no)** | 0.66 | [0.50, 0.87] | 0.003 |

**Supplemental table S2.** Multivariate linear model on growth velocity (g/kg/day).

| **Predictor** | **Estimate (β)** | **95% CI** | **p-value** |
| --- | --- | --- | --- |
| **Probiotics (Yes vs No)** | 1.80 | [0.93, 2.67] | <0.001 |
| **Donor Human Milk (vs MOM)** | –2.27 | [–3.58, –0.95] | 0.001 |
| **Formula (vs MOM)** | 0.94 | [–1.59, 3.47] | 0.467 |
| **Sex (Male vs Female)** | –0.38 | [–1.23, 0.46] | 0.378 |
| **NEC/LOS/mortality (yes vs no)** | –0.99 | [–1.89, –0.08] | 0.033 |
| **AGA (vs SGA)** | –5.20 | [–6.61, –3.79] | <0.001 |
| **LGA (vs SGA)** | –7.67 | [–9.75, –5.60] | <0.001 |
